# Differences in Cardiovascular Manifestation of Marfan Syndrome Between Children and Adults

**DOI:** 10.1007/s00246-018-2025-2

**Published:** 2018-11-11

**Authors:** L. Wozniak-Mielczarek, R. Sabiniewicz, M. Drezek-Nojowicz, R. Nowak, N. Gilis-Malinowska, M. Mielczarek, A. Łabuc, A. Waldoch, J. Wierzba

**Affiliations:** 1 Department of Pediatric Cardiology and Congenital Heart Diseases, Medical University of Gdansk, Gdansk, Poland; 2 Department of Ophthalmology, Medical University of Gdansk, Gdansk, Poland; 3 2nd Department of Cardiology, Medical University of Gdansk, Gdansk, Poland; 4 1st Department of Cardiology, Medical University of Gdansk, Gdansk, Poland; 5 Department of Orthopaedics and Motor Organ Traumatology, Medical University of Gdansk, Gdansk, Poland; 6 Department of Nursery, Medical University of Gdansk, Gdansk, Poland

**Keywords:** Marfan syndrome, Aortic root dilatation, Aortic dissection, Mitral valve regurgitation, Mitral valve prolapse

## Abstract

Marfan syndrome (MFS) is a connective tissue disorder characterized by a broad range of clinical manifestations. Cardiovascular involvement is the most life-threatening aspect of the syndrome. Although abnormalities within the cardiovascular system in adults are well documented, there is still a paucity of data regarding manifestation of MFS in childhood. The aim of the study was to compare cardiovascular manifestation of MFS between children and adults. The study population consisted of 236 patients (144 children and 92 adults), who were referred to our department with suspicion of MFS. All patients underwent complete clinical evaluation in order to confirm the diagnosis of MFS according to the modified Ghent criteria. MFS was diagnosed in 101 (44 children and 57 adults) out of the 236 patients. The other patients were diagnosed with Ehlers-Danlos syndrome, Loeys–Dietz syndrome, MASS phenotype, ectopia lentis syndrome, marfanoid habitus and other rare syndromes. The most common cardiovascular abnormality was aortic root dilatation (81.19% of patients). It was found that both adults and children had similar high rates of aortic root dilatation. Similarly, there was no significant difference with regard to the prevalence of aortic valve regurgitation and mitral valve prolapse among children and adults. These findings equivocally indicate that the aforementioned abnormalities develop in early childhood, therefore, they may be used in the early identification of patients with MFS. Other assessed abnormalities, which included mitral valve regurgitation, pulmonary artery dilation, aneurysms of aortic arch, descending thoracic aorta and abdominal aorta were found mostly in adults, and thus, are of less use in the early detection of MFS.

## Introduction

Marfan syndrome (MFS) is an autosomal dominant genetic disorder of connective tissue caused most frequently by mutations in the fibrillin-1 gene [1, 2]. It is characterized by a broad range of clinical manifestations mainly involving not only the skeletal, ocular and cardiovascular system, but also adipose and muscle tissue, skin, pulmonary and central nervous system [3–5]. Cardiovascular involvement in the form of aortic aneurysm and aortic dissection or rupture is the most life-threatening aspect of the syndrome [6–8]. Other cardiovascular findings associated with MFS include aortic regurgitation, mitral valve prolapse and regurgitation, tricuspid valve prolapse and regurgitation, pulmonary artery dilatation and primary cardiomyopathy [6, 9–11]. Furthermore, existing data suggest increased prevalence of ventricular arrhythmia and long QT syndrome [12, 13]. Although abnormalities within the cardiovascular system in adults are quite well known, there is still a paucity of data regarding manifestation of MFS in childhood. It is particularly unclear whether there are differences in the involvement of the cardiovascular system between adolescence and adulthood. The aim of the study was to evaluate the cardiovascular system in children and adults with MFS and to compare the type, incidence and severity of these findings between the two groups.

## Materials and Methods

### Patients

Between January 2015 and January 2018, 236 patients (144 children and 92 adults) aged 2 months to 65 years were referred with suspicion of MFS. The most common reason for MFS suspicion was a very tall and slim silhouette (26.85%), followed by the presence of MFS in the family (23.96%), joint hypermobility (13.62%), chest deformity (6.61%), scoliosis (5.84%), aortic dilatation or dissection (5.45%) and lens dislocation (5.45%). All patients underwent complete clinical assessment including detailed medical history (with family medical history), physical examination with anthropometrics measurements, cardiac examination (electrocardiography, 24-h ambulatory electrocardiographic monitoring and transthoracic echocardiography) as well as ophthalmologic, orthopedic and genetic consultations. Finally, the modified Ghent criteria was used to identify patients with MFS [14]. The study was approved by the local Ethics Committee.

### Echocardiography

**Fig. 1:**
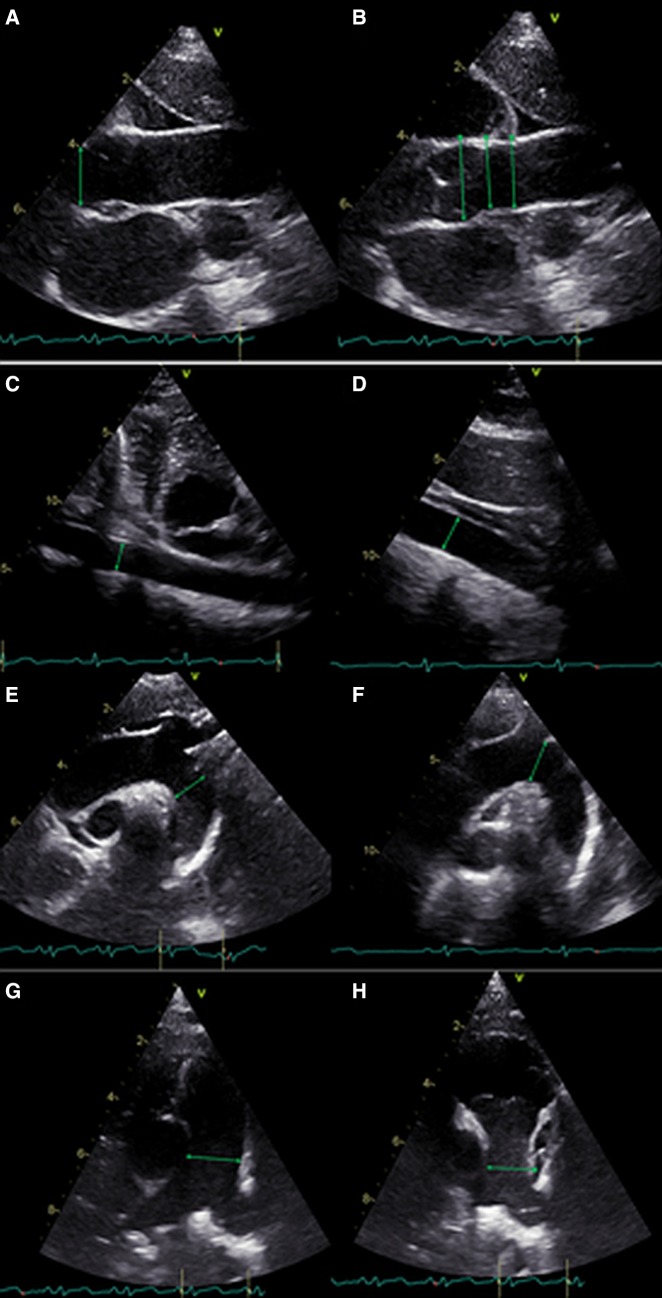
Overview of applied techniques for dimensioning segments of the aorta and the pulmonary trunk in children and adult population. **a** aortic annulus (inner edge in mid-systole according to Gautier et al. and Roman et al.); **b** aortic root, sinotubular junction, distal ascending aorta (leading edge in end-diastole, Gautier et al. and Devereux et al.); **c** thoracic descending aorta (inner edge in mid-systole, Pettersen et al. and Roman et al.); **d** abdominal aorta (inner edge in mid-systole, Pettersen et al. and Evangelista et al.); **e** aortic arch (inner edge in end-diastole, Mirea et al.); **f** aortic arch (inner edge in mid-systole, Pettersen et al.); **g** pulmonary trunk (inner edge in mid-systole, Pettersen et al.); **h** pulmonary trunk (leading edge in end-diastole, Sheikhzadeh et al.)

Transthoracic echocardiography (TTE) was performed using Vivid E95 and Vivid S6 ultrasound system and M5Sc or 6S transducers manufactured by General Electric. Each echocardiogram was conducted by an experienced cardiologist in accordance with the recommendations of the European Association of Cardiovascular Imaging (EACVI) and the American Society of Echocardiography (ASE) [15]. Special attention was paid to aortic diameters measured within the ascending aorta (at the level of the aortic annulus, aortic root, sinotubular junction and distal ascending aorta), aortic arch, descending thoracic aorta and abdominal aorta as well as pulmonary trunk diameter. In children, all dimensions were expressed in *z-score* that incorporates body surface area (BSA) and sex [16, 17]. In adults, special *z-*score calculators were used to correct aortic root diameter for BSA, sex and age [18, 19]. The *z-*score describes how many standard deviations are above or below mean predicted diameter for the examined patient [20, 21]. Aortic dilatation was confirmed when the *z-*score was ≥ 2. Detailed technique of aortic and pulmonary trunk diameter measurements as well as norms used for particular age groups are presented in Tables 1 and 2 and illustrated in Fig. 1. Measurement methods were selected based on available literature data. Aortic arch and pulmonary main artery measurement techniques were different in children and adults. This was unavoidable because nomograms for children and adults are based on different methodology. For this reason, we didn’t compare nominal values of diameters, but only the final results (dilated or not dilated). For most calculations, we included all patients enrolled into the study. However, there was a rationale in some analyses to perform calculations only on patients who hadn’t had prior surgery on ascending aorta (i.e. aortic root diameter; aortic annulus, STJ and distal ascending aorta dilatation and diameter; aortic valve regurgitation).

**Table 1:** Applied techniques and norms for dimensioning segments of the aorta and the pulmonary trunk in child population

Artery segment	Echocardiographic projection	Measurement technique	Applied standards
Aortic annulus	Parasternal long axis view	Inner edge in mid-systole, maximal diameter at the hinge points of the leaflets	Gautier et al. [16]
Aortic root		Leading edge in end-diastole, the largest diameter within the sinuses of Valsalva	
Sinotubular junction		Leading edge in end-diastole, maximal diameter at the transition point from sinus to tubular aorta	
Distal ascending aorta		Leading edge in end-diastole, maximal diameter 1 cm behind sinotubular junction	
Aortic arch	Suprasternal view	Inner edge in mid-systole, maximal dimension between the innominate and left common carotid arteries	Pettersen et al. [17]
Descending thoracic aorta	Modified apical four chamber view	Inner edge in mid-systole, maximal dimension in the middle part of thoracic aorta	
Abdominal aorta	Subcostal view	Inner edge in mid-systole, maximal dimension at the level of the diaphragm	
Pulmonary main artery	Parasternal short axis view	Inner edge in mid-systole, maximal dimension, halfway between the pulmonary valve and the split of the pulmonary trunk on the branches	

**Table 2:** Applied techniques and norms for dimensioning segments of the aorta and the pulmonary trunk in adult population

Artery segment	Echocardiographic projection	Measurement technique	Applied standards
Aortic annulus	Parasternal long axis view	Inner edge in mid-systole, maximal diameter at the hinge points of the leaflets	Roman et al. [18]
Aortic root		Leading edge in end-diastole, the largest diameter within the sinuses of Valsalva	Devereux et al. [19]
Sinotubular junction		Leading edge in end-diastole, maximal diameter at the transition point from sinus to tubular aorta	Roman et al. [18]
Distal ascending aorta		Leading edge in end-diastole, maximal diameter 1 cm behind sinotubular junction	Roman et al. [18]
Aortic arch	Suprasternal view	Inner edge in end-diastole, maximal dimension perpendicular to the blood flow at the side of the distal wall of the left subclavian artery	Mirea et al. [22]
Descending thoracic aorta	Modified apical four chamber view	Inner edge in mid-systole, maximal dimension in the middle part of thoracic aorta	Evangelista et al. [23]
Abdominal aorta	Subcostal view	Inner edge in mid-systole, maximal dimension at the level of the diaphragm	Evangelista et al. [23]
Pulmonary main artery	Parasternal short axis view	Leading edge in end-diastole, maximal dimension, halfway between the pulmonary valve and the split of the pulmonary trunk on the branches	Sheikhzadeh et al. [24]

### Statistical Analysis

Continuous data were presented as a mean value and standard deviation (SD), while categorical data were presented as percentages. Normal distribution was verified by Kolmogorov–Smirnov test. Continuous data were compared using the Student *t* test or the U Mann Whitney test depending on the distribution or in case of comparing more than two groups by univariate analysis of variance (ANOVA) followed by the LSD test (Least Significant Difference) or by the Kruskall–Wallis test. Categorical data were compared using Chi square test and Fisher’s exact test when appropriate. Correlation between two continuous variables was performed using Pearson correlation. A *p* value of < 0.05 was considered statistically significant. Data were analyzed using SPSS software v.21 (IBM, Chicago, Illionois, USA).

## Results

### Patient Characteristics

Out of the 236 patients examined, MFS was confirmed in 101 patients (44 children and 57 adults) in accordance to the Ghent criteria. In addition, two infants were diagnosed with neonatal Marfan syndrome, but because of its different clinical course, they were excluded from the study [25, 26]. The other patients were diagnosed with Ehlers–Danlos syndrome (*n* = 25), Loeys–Dietz syndrome (*n* = 7), MASS phenotype (*n* = 3), ectopia lentis syndrome (*n* = 2), marfanoid habitus—external features of MFS (*n* = 89) and other rare syndromes (*n* = 7). The mean age in the MFS group was 23.76 ± 15.32 years (from 2 months to 65 years) and 46 (45.54%) were female. Sixty-nine (68.32%) individuals had a positive family history for MFS, while 32 (31.68%) most likely had sporadic mutation. The patients with a positive family history for MFS belonged to 39 families.

### Aortic Root

**Fig. 2:**
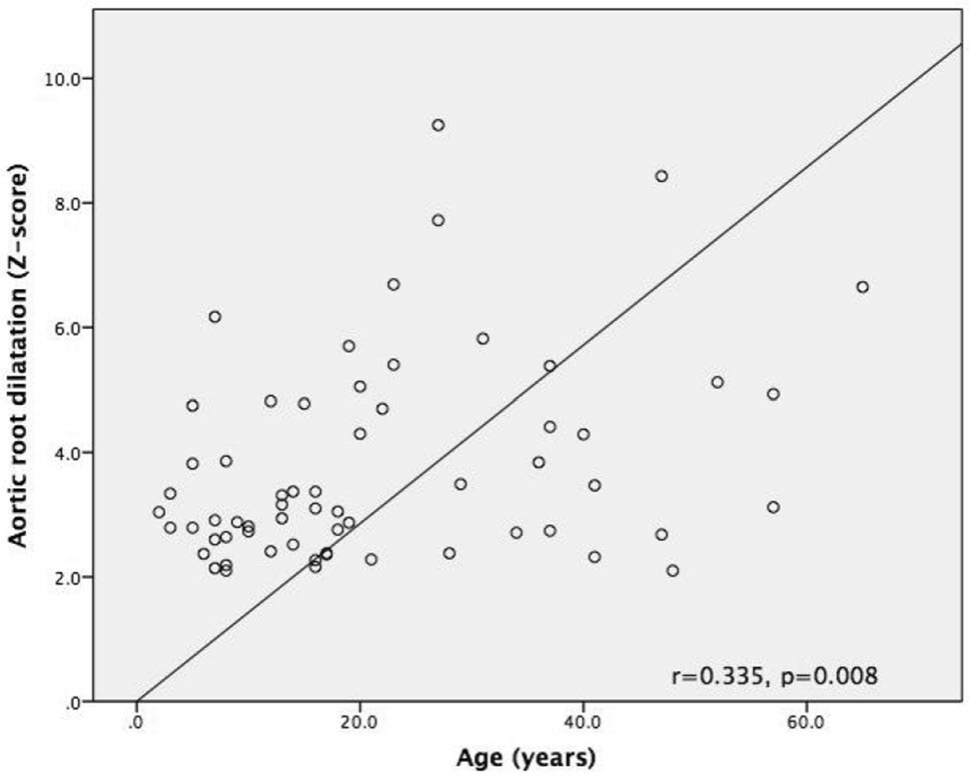
Diagram presenting Pearsons correlation between aortic root diameter (*z*-score) and patient age (years), *r* = 0.335, *p* = 0.008

In children with MFS, aortic root diameters ranged from − 0.22 to + 6.17 *z*-score (mean 2.57 ± 1.26) and from 17 to 46 mm (mean 31.54 ± 6.50). In the adult MFS, subset aortic root diameters ranged from + 0.31 to + 9.25 *z*-score (mean + 3.73 ± 2.22) and from 31 to 60 mm (mean 42.15 ± 6.53). Overall, aortic root dilatation (*z*-score ≥ 2) was diagnosed in 82 (81.19%) patients. There were 33 children (75%) and 49 adults (85.96%) with aortic root dilatation, including patients after surgery for aortic root aneurysms. There was no significant difference with regard to the prevalence of aortic root dilatation between children and adults (*p* = 0.202). Moreover, 4 children (9.09%) and 1 adult (1.75%) had aortic root diameter at the upper limit of the normal range (*z*-score from 1.9 to 1.99). Among all the patients with a dilated aortic root, the mean aortic root diameter was 3.75 ± 1.63 *z*-score (3.09 ± 0.93 *z*-score in children and 4.46 ± 1.91 *z*-score in adults). Aortic root dilatation was significantly larger in adults than in children (*p* = 0.001). Detailed analysis (Pearson correlation) demonstrated a linear correlation between aortic root diameter and patient age (*r* = 0.335, *p* = 0.008) (Fig. 2).

**Fig. 3:**
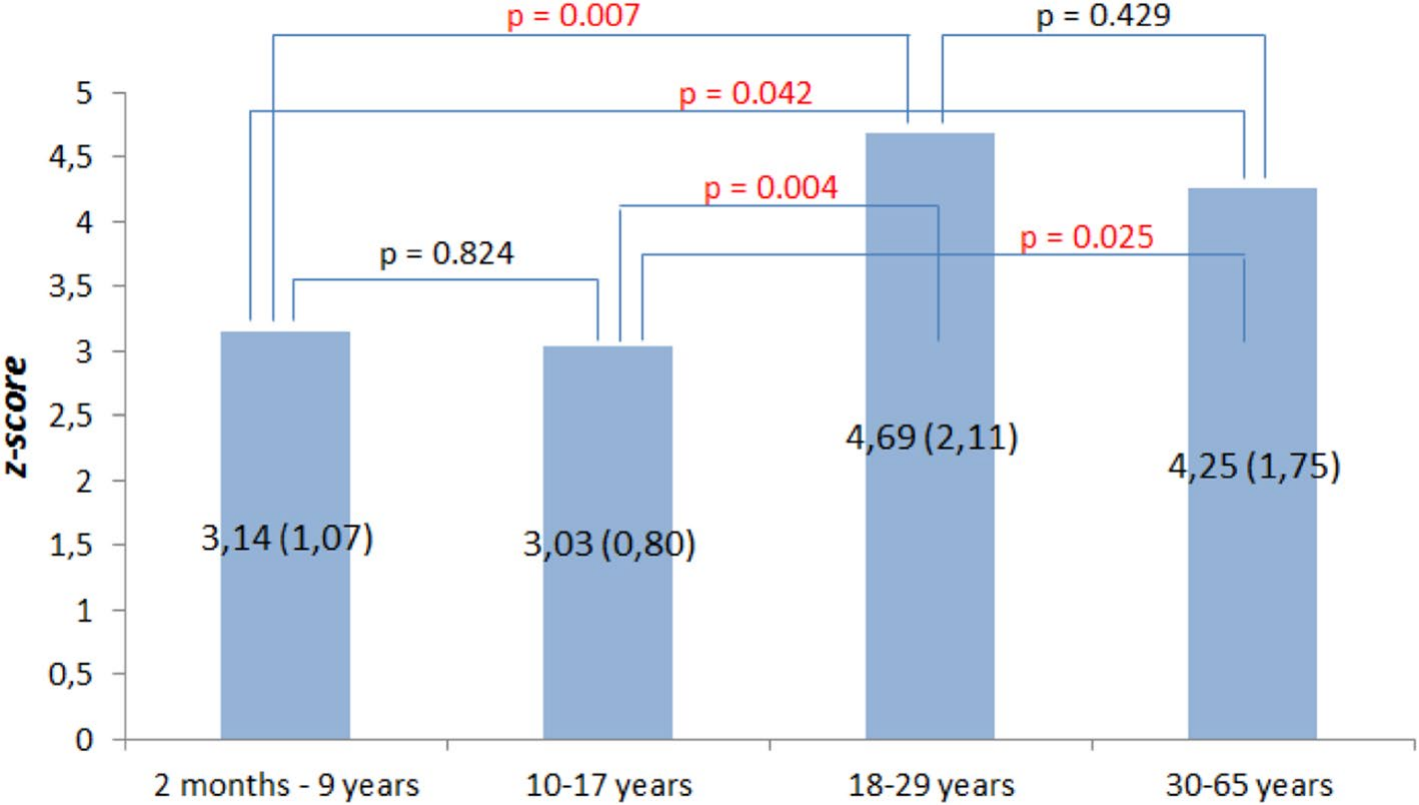
Mean aortic root diameters (*z*-score) in patients with aortic root dilatation divided into four categories according to age and their comparison in different age categories (statistically significant differences were marked in red); standard deviations were presented in brackets

Finally, patients with aortic root dilatation were divided into four categories according to age: 2 months to 9 years, 10–17 years, 18–29 years and 30–65 years. Significant differences between these groups were noted with regard to aortic root dilatation (*p* = 0.007). The largest aortic root dilatation was observed in patients between 18 and 29 years old (Fig. 3).

Furthermore, we analyzed differences in the prevalence of aortic root dilatation between male and female patients; however, no significant gender differences were found in this regard both in adults (82.76% in women vs. 89.29% in men, *p* = 0.706) and in children (64.71% in girls vs. 81.48% in boys, *p* = 0.289). Similarly, there were no gender differences with regard to the degree of aortic root dilatation (expressed exceptionally in mm/BSA in order to disregard the sex coefficient, which is included in *z*-score calculators) both in adults (23.34 ± 3.46 in women vs. 22.02 ± 2.53 in men, *p* = 0.234) and in children (28.26 ± 5.44 in girls vs. 27.30 ± 6.24 in boys, *p* = 0.669).

### Other Segments of the Ascending Aorta

**Fig. 4:**
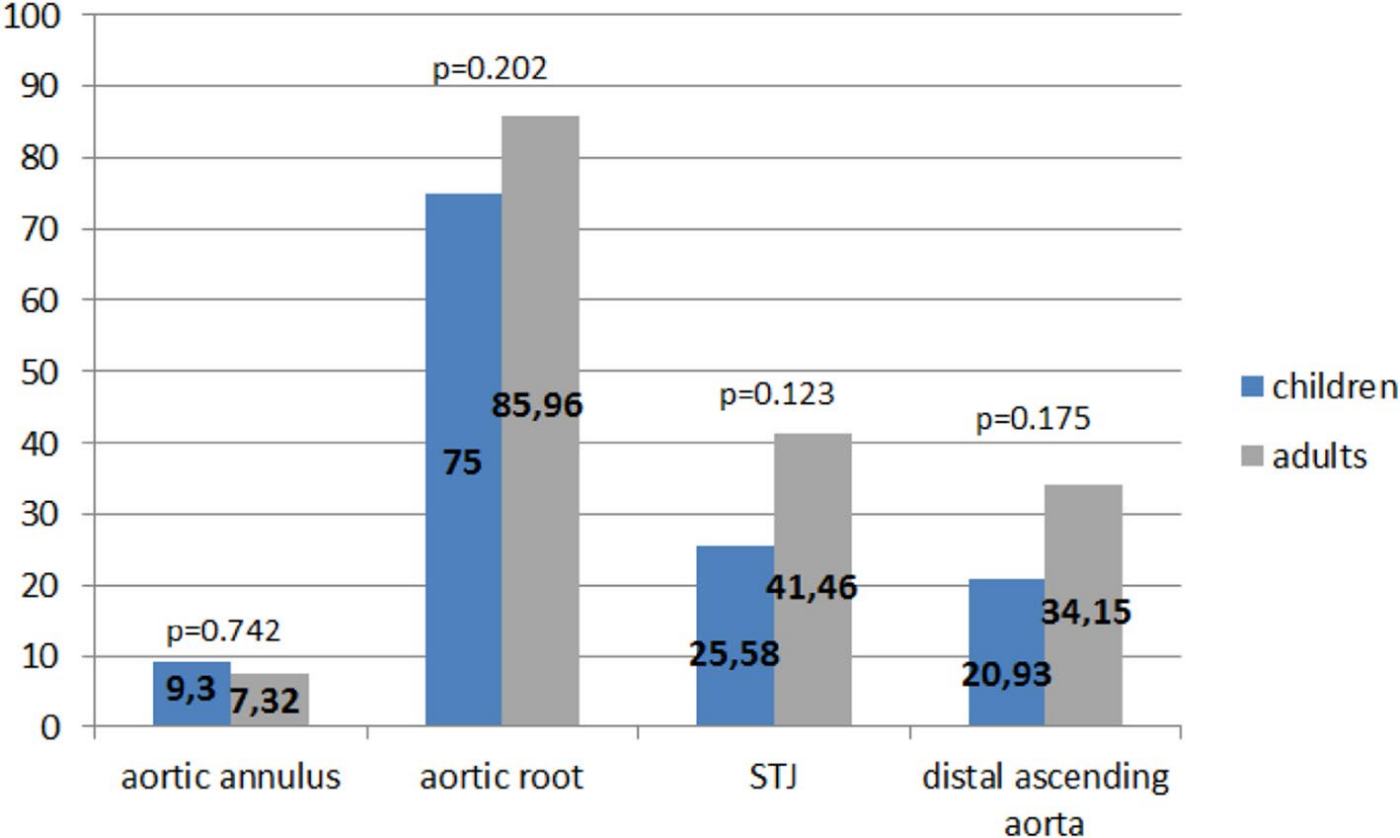
Diagram presenting comparison of prevalence (percentage of child or adult subset) of dilatation of all ascending aorta segments: aortic annulus, aortic root, STJ and distal ascending aorta between children and adults with MFS

The other segments of the ascending aorta were affected much less than the aortic root. Dilatation of the aortic annulus was found only in 7 (8.33%) patients with MFS, sinotubular junction (STJ) dilatation in 28 (33.33%) patients and distal ascending aorta in 23 (27.38%) patients. The above listed segments of the aorta were affected with similar prevalence in children and adults, *p* = 0.742, *p* = 0.123, *p* = 0.175, respectively. All cases of aortic annulus dilatation were mild, mean 2.74 ± 1.12 *z*-score. The mean STJ diameter was + 3.45 ± 2.01 *z*-score. The mean distal ascending aorta diameter was + 3.19 ± 2.06 *z*-score (Table 3). In all patients with dilatation of the aortic annulus, STJ or distal ascending aorta, the aortic root was also affected. In five patients, dilatation of the distal ascending aorta was larger than aortic root dilation, but only in one patient, who additionally had a bicuspid aortic valve, was the difference significant: + 2.27 *z*-score (Fig. 4).

**Table 3:** Comparison of the most common cardiovascular system abnormalities between children and adults with MFS

	All patients (*n* = 101)	Children (*n* = 44)	Adults (*n* = 57)	*p* (children vs. adults)
Aortic root dilatation (%)	81.19	75	85.96	0.202
Aortic arch dilatation (%)	8.91	2.27	14.04	**0.040**
Descending thoracic and abdominal aorta dilatation (%)	10.89	2.27	17.54	**0.015**
Aortic dissection (%)	7.92	4.55	10.53	0.270
Type A aortic dissection (%)	4.95	4.55	5.26	0.869
Type B aortic dissection (%)	2.97	0	5.26	0.122
Pulmonary trunk dilatation (%)	27.72	15.91	36.84	**0.025**
Bicuspid aortic valve (%)	3.96	6.82	1.75	0.196
Mitral valve regurgitation (%)	63.37	45.45	77.19	**0.002**
Severe mitral valve regurgitation (%)	9.90	4.55	14.04	0.113
Mitral valve prolapse (%)	54.46	56.82	52.63	0.692
Frequent ventricular extrasystoles (%)	8.91	4.55	12.28	0.176
Frequent supraventricular extrasystoles (%)	4.95	4.55	5.26	0.869

	Patients without prior surgery on ascending aorta (*n* = 84)	Children (*n* = 43)	Adults (*n* = 41)	*p* (children vs. adults)
Aortic root diameter^a^ (*z*-score)	+ 3.75 ± 1.63	+ 3.09 ± 0.93	+ 4.46 ± 1.91	**0.001**
Aortic annulus dilatation (%)	8.33	9.30	7.32	0.742
Aortic annulus diameter^a^ (*z*-score)	+ 2.74 ± 1.12	+ 2.66 ± 0.22	+ 2.67 ± 1.68	0.969
STJ dilatation (%)	33.33	25.58	41.46	0.123
STJ diameter^a^ (*z*-score)	+ 3.45 ± 2.01	+ 3.02 ± 1.98	+ 3.74 ± 1.75	0.056
Distal ascending aorta dilatation (%)	27.38	20.93	34.15	0.175
Distal ascending aorta diameter^a^ (*z*-score)	+ 3.19 ± 2.06	+ 3.16 ± 1.43	+ 3.22 ± 1.65	0.848
Aortic valve regurgitation (%)	30.95	25.58	36.59	0.490

Statistically significant differences are marked in bold

^a^Among patients with dilation of this part of aorta

### Aortic Arch

In total, aortic arch dilatation was found in 9 (8.91%) patients. Prevalence of aortic arch dilatation was significantly higher in adults than in children (*p* = 0.040). Two adults required surgery for aortic arch aneurysms. Other patients had mild to moderate aortic arch dilatation.

### Descending Thoracic and Abdominal Aorta

Overall, descending thoracic or abdominal aorta dilatation was found in 11 (10.89%) patients with higher prevalence in adults than children (*p* = 0.015). Eight (1 child and 7 adults) patients had thoracoabdominal aneurysm, which required surgery. The other two patients had moderate isolated abdominal aorta dilatation, while one patient had mild dilatation of thoracoabdominal aorta.

### Aortic Dissection

In retrospective data analysis, aortic dissection was noted in 8 (7.92%) patients: 6 adults (10.53%) and 2 children (4.55%), *p* = 0.270. It occurred at the mean age of 26.17 ± 6.96 years (from 16 to 35). Type A aortic dissection according to Stanford classification was diagnosed in five patients. Analysis of CT angiography in patients with type A aortic dissection revealed that the mean diameter of the aortic root (false and real lumen together) at the time of event was 51.8 ± 7.49 mm (mean + 7.51 *z*-score). Importantly, in three out of four women, aortic dissection occurred during the third trimester of pregnancy. Both children who experienced aortic dissection were teenagers, in the age of 16 and 17 years. The first one was diagnosed with aortic root aneurysm of 52 mm in diameter. That patient experienced aortic dissection, while waiting for elective aortic root surgery and died before any medical help due to massive hemothorax. The second child, who hadn’t had the diagnosis of MFS before, experienced aortic dissection during volleyball game. His aortic root diameter was 60 mm, which was assessed during emergent surgery.

### Pulmonary Trunk

The pulmonary trunk was considered dilated, when its diameter measured ≥ 2 *z-*score in children and ≥ 27 mm in adults (Tables 1 and 2). Pulmonary trunk dilatation was noted in 28 (27.72%) patients and it was more often in adults than in children: 21 (36.84%) versus 7 (15.91%), respectively, *p* = 0.025. In children with MFS, pulmonary trunk diameter ranged from − 0.85 to + 2.55 *z*-score (mean + 1.03 ± 0.91 *z*-score), while in adults from 18 to 41 mm (mean 29.31 ± 5.70 mm). None of the patients required surgical intervention.

### Aortic Valve

Bicuspid aortic valve was present in 4 (3.96%) patients with no significant difference with regard to the prevalence between adults and children (*p* = 0.196). All the analysis regarding aortic valve regurgitation was confined to patients without prior surgery on the aortic valve or ascending aorta. Aortic valve regurgitation was diagnosed in 26 (30.95%) patients, with similar prevalence being noted in adults and children (*p* = 0.490). In 2 adults, aortic valve regurgitation was considered moderate. In the other 24 patients (92.31%), it was mild.

### Mitral Valve

Mitral valve regurgitation was found in 64 patients (63.37%) and it was much more common in adults than in children (*p* = 0.002). Among the children, 2 (10%) had severe regurgitation, 3 (15%) had moderate and the other 14 (75%) mild. In the adult subset, severe mitral valve regurgitation was recognized in 8 (18.18%) patients, moderate in 32 (72.73%) patients and mild in 4 (9.09%) patients. Altogether, mitral valve prolapse was present in 55 patients (54.46%). Its prevalence was similar in the adult and child subsets (*p* = 0.692). In the other 9 (8.91%) patients with mitral regurgitation without mitral valve prolapse, valve insufficiency was regarded mild without specified etiology.

### Cardiac Surgeries

**Fig. 5:**
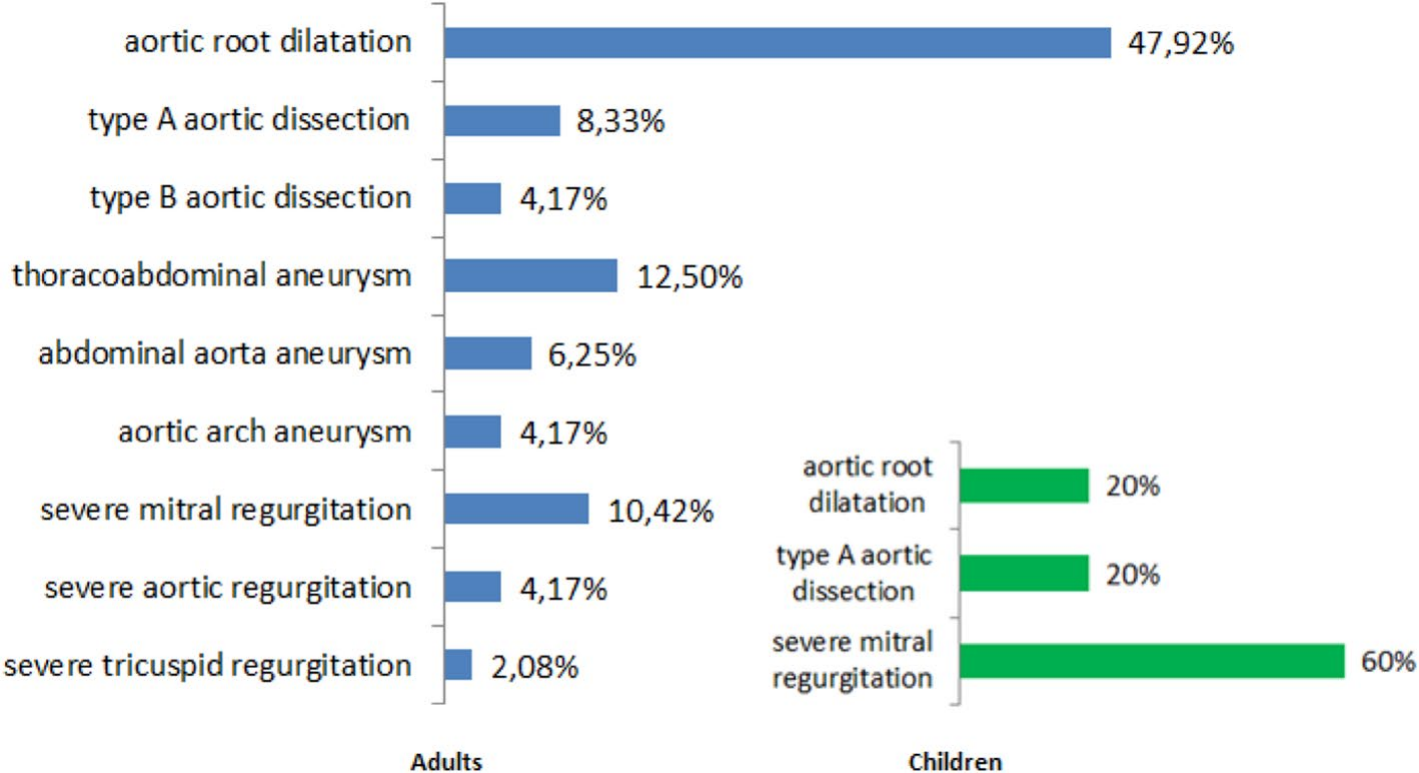
List of reasons for cardiac surgery qualification in patients with MFS

Among the 101 patients with MFS, 34 (33.66%) underwent 53 cardiac surgeries (operations conducted due to perioperative complications or coexisting diseases were not included). The most common indication for surgery was aortic root dilatation (45.28% of all surgeries). In those cases, valve sparing aortic root replacement (David procedure) prevailed (54.16%) over Bentall–de Bono procedure consisting of composite graft replacement (45.83%). Other reasons for cardiac surgeries were severe mitral regurgitation (15.09%), thoracoabdominal aneurysm (11.32%), Stanford-type A aortic dissection (9.43%), abdominal aorta aneurysm (5.66%), Stanford-type B aortic dissection (3.77%), severe aortic regurgitation (3.77%), aortic arch aneurysm (3.77%) and severe tricuspid regurgitation (1.89%) (Fig. 5). The mean age at the time of the first surgery was 28.45 ± 9.93 years. Five patients had their first surgery during childhood. The most common indication for cardiac surgery during childhood was severe mitral regurgitation (three patients), other reasons were aortic root dilatation (one patient) and Stanford-type A aortic dissection (one patient).

### Other Echocardiography Findings

Among the patients examined for MFS, there were also some with rare cardiovascular system abnormalities. In 2 (2.99%) patients, left ventricular contractility was significantly impaired (patients after cardiac surgery were excluded from these calculations). One (0.99%) patient had undergone an anatomic correction of transposition of the great arteries and another (0.99%) surgical repair of a vascular ring anomaly (double aortic arch). In 2 (1.98%) patients, a coronary artery fistula to the pulmonary trunk (hemodynamically insignificant) was detected. Three (2.97%) patients had atrial septal defect, 1 (0.99%) ventricular septal defect, 6 (5.94%) tricuspid valve prolapse and 2 (1.98%) a narrowing of the orifice of the tricuspid valve without blood flow impairment (both patients had severe pectus excavatum).

### Electrocardiography and 24-h Ambulatory Electrocardiographic Monitoring

Furthermore, electrocardiography (ECG) and 24-h ambulatory electrocardiographic monitoring (Holter ECG) recording was analyzed. Among all the MFS patients, an increased number of ventricular extra beats (over 1000/day) were detected in 9 (8.91%) of them, with similar prevalence in adults and children, 7 (12.28%) versus 2 (4.55%), respectively, *p* = 0.176. An increased number of supraventricular premature contractions (over 1000/day) were noted in five patients with MFS (4.95%), with similar prevalence in adults and children, 3 (5.26%) versus 2 (4.55%), respectively, *p* = 0.869. In addition, in the adult MFS subset, 2 (3.51%) patients had a pacemaker implanted due to postoperative third-degree atrioventricular block and 1 (1.75%) patient had a cardioverter-defibrillator (ICD) implanted as a secondary prevention of ventricular fibrillation. In the child subset, 1 (2.23%) patient had prolonged QT interval.

## Discussion

To the best of our knowledge, this is the first study to directly compare abnormalities in the cardiovascular system between children and adults with MFS. In general, the most common cardiovascular abnormality in both age groups was aortic root dilatation. Similar rates (from 65.2 to 88.6%) of aortic root dilatation have been shown by other authors [6, 9, 16, 27–31]. In the present study, it was found that both adults and children had similar high rates of aortic root dilatation, which indicated that the majority of patients developed aortic root dilatation in early childhood. Furthermore, we documented that BSA corrected aortic root diameter increases with the patient’s age. Importantly, the largest aortic root dilatation was found in patients between 18 and 29 years old. It seems that the smaller diameter of the aorta in patients between 30 and 65 years old might be associated with previously performed cardiac surgeries on the aortic root and deaths due to acute aortic syndrome that occurred at a younger age. These findings are of great clinical importance because they indicate, that relatively young patients should already be under systematic guidance and be referred for aortic root surgery once echocardiographic criteria are met.

Even though literature data have shown that women with MFS live up to 10 years longer than men with MFS [32, 33], we did not find any significant gender differences with regard to prevalence of aortic root dilatation and aortic root diameter in both adults and children. Similar results also expressed in mm/BSA were published by Roman et al. [28]. On the other hand, Meijboom et al. have shown that aortic root diameter in women with MFS expressed in mm is significantly smaller, even if corrected with BSA [34]. It may be speculated that men more often perform jobs that require isometric physical effort. However, further research is needed to elucidate the reason for shorter life among men with MFS.

It was found that in both age groups other segments of the ascending aorta (aortic annulus, STJ, distal ascending aorta) were dilated much less often than the aortic root. Notwithstanding, dilation of other segments of the ascending aorta (distal ascending aorta) expressed in *z*-score was significantly larger than the dilation of the aortic root in only one patient—it was a patient with coexisting bicuspid aortic valve.

In contrast to aortic root dilation, aneurysms of the aortic arch, descending thoracic aorta and abdominal aorta were much more common in adults than in children. However, we found that occasionally they do occur in children, especially in teenagers. Furthermore, even though there was a tendency toward greater prevalence of aortic dissections in adults than in children, 2 teenagers (16 and 17 year-old) from the studied group had experienced aortic dissection. These findings clearly document the need for careful assessment of the entire aorta in patients with MFS in all ages.

In the studied group, three women experienced aortic dissection during pregnancy, despite the fact that in all of them the aortic root was only mildly widened. It should be said that none of them were diagnosed with MFS earlier, therefore, they didn’t get any particular recommendations considering their status.

In our population, pulmonary trunk dilatation was found in 37% of adults. This was significantly less often than what was noted in two other studies dealing with pulmonary artery dilatation in patients with MFS: Nollen et al.—reported an prevalence of 74% and Sheikhzadeh et al.—69.4% [10, 24]. In our study, children rarely presented with pulmonary trunk dilatation (15.91%) compared to adults. There are no previous data regarding the prevalence of pulmonary trunk dilatation in children with MFS. None of the patients required surgery for pulmonary trunk dilatation, which is in accordance with previous studies. There is only one case report describing the rupture of pulmonary artery aneurysm in a patient with MFS [35]. Even though the histological structure of the pulmonary trunk is similar to that of the aorta, it widens much less often, most likely due to lower pressure in the pulmonary circulation.

The second most common cardiovascular system abnormality, after aortic root dilatation, in patients with MFS in our population was mitral valve regurgitation. It was found in 63.37% of patients, significantly more often in adults than in children. In the majority of patients, the cause of regurgitation was mitral valve prolapse. Mitral valve prolapse was found in about half of patients, with a similar prevalence in all the age groups. Similar high rates of mitral valve prolapse were reported by Pyeritz et al. (68%), Roman et al. (62% of children and 55% of adults) and Faivre et al. (60.36% of children) [28, 31, 36]. On the other hand, it should be mentioned that there are studies showing either much greater prevalence of mitral valve prolapse—88.5% (Karnebeek et al.), 100% (Geva et al.) and 100% (Ozdemir et al.) or much lower—17.5% (Lipscomb et al.) and 29% (Lima et al.), which indicates that the mitral valve prolapse diagnostic criteria is equivocal [6, 27, 30, 37, 38]. Most of the previous studies evaluated the prevalence of mitral valve prolapse, but not mitral valve regurgitation. This is due to the fact that the latter isn’t included in the modified Ghent criteria. In three out of four available studies, a very high prevalence of mitral valve regurgitation in patients with MFS was reported: from 81 to 100% [6, 30, 37]. Only in the paper by Lipscomb et al. was the prevalence of mitral regurgitation surprisingly low—12.77% [38].

Although aortic valve regurgitation is described as one of the main abnormalities in patients with MFS syndrome, in our study it was found only in 30.59% patients—similarly in adults and children, and in the vast majority of patients it was categorized as mild. Only in two patients was aortic valve regurgitation considered moderate, none of the patients had severe aortic valve regurgitation. Similar prevalence of aortic valve regurgitation (31.86%) was reported by Roman et al. [28], but in contrast to our study, in a large proportion of patients (25%), it was categorized as severe. In several other studies, which assessed children with MFS, the prevalence of aortic regurgitation was similar or lower—Karnebeek et al. (25%), Geva et al. (28%), Ozdemir et al. (18.18%) and Lipscomb et al. (2.5%) [6, 30, 37, 38].

Cardiac surgeries are rather inescapable in patients with MFS. In our study, 54.39% of adults and 6.82% of children underwent at least one cardiac surgery. In our group, the mean age at the time of first surgery was 28.45 ± 9.93 years. In the vast majority of patients, the surgery was carried out due to aortic root dilatation or severe mitral regurgitation. There is paucity of data, on how many patients with MFS require cardiac surgery. Lima et al. reported a similar rate of 62% in a population with MFS above 15 years old, with the mean age at the time of first surgery of 35.5 ± 11.3 years [27]. Karnebeek et al. assessed cardiac surgeries in children with MFS and reported its prevalence at 26% [6]. In both the abovementioned studies, the reasons for the surgeries were similar to our research.

## Limitations

The study has several limitations that need to be pointed out. First, the study was planned as a prospective registry, based on voluntary referrals. Therefore, the possibility of referral bias couldn’t be excluded. Second, we present data collected during a single visit, without providing any longitudinal data on patients follow-up. Third, genetic testing was not performed in patients diagnosed with MFS based on clinical criteria. Importantly, genetic testing isn’t obligatory to make a diagnosis of MFS, nevertheless, it might have allowed us to determine if there was a connection between a particular genetic mutation and cardiovascular manifestation.

## Conclusions

According to the presented study, the most common cardiovascular abnormalities in patients with MFS are as follows: aortic root dilatation, mitral valve regurgitation, mitral valve prolapse, aortic valve regurgitation and pulmonary artery dilatation. The study revealed, that the prevalence of aortic root dilatation, aortic valve regurgitation and mitral valve prolapse are similar among children and adults. These findings suggest that the aforementioned abnormalities develop in early childhood and simply progress with the process of time. Therefore, they may be used in the early identification of patients with MFS. The other assessed abnormalities, which were mitral valve regurgitation, pulmonary artery dilatation, aneurysms of aortic arch, descending thoracic aorta and abdominal aorta were found mostly in adults, thus, are of less use in early identification of MFS. Importantly, the largest BSA corrected aortic root dilatation was found in patients between 18 and 29 years old. Smaller diameter of the aorta in patients between 30 and 65 years old might be associated with previously performed cardiac surgeries on the aortic root and deaths due to acute aortic syndrome that occurred at a younger age. These findings are of great clinical significance because they indicate, that relatively young patients should already be under systematic guidance and be referred for aortic root surgery once echocardiographic criteria are met. Cardiac surgeries are rather inescapable in patients with MFS, but as our analysis showed, in the vast majority of patients the indications for surgery appear in adulthood.
